# Bee Venom—A Potential Complementary Medicine Candidate for SARS-CoV-2 Infections

**DOI:** 10.3389/fpubh.2020.594458

**Published:** 2020-12-10

**Authors:** Keneth Iceland Kasozi, Gniewko Niedbała, Mohammed Alqarni, Gerald Zirintunda, Fred Ssempijja, Simon Peter Musinguzi, Ibe Michael Usman, Kevin Matama, Helal F. Hetta, Ngala Elvis Mbiydzenyuy, Gaber El-Saber Batiha, Amany Magdy Beshbishy, Susan Christina Welburn

**Affiliations:** ^1^Infection Medicine, Deanery of Biomedical Sciences, College of Medicine and Veterinary Medicine, The University of Edinburgh, Edinburgh, United Kingdom; ^2^School of Medicine, Kabale University, Kabale, Uganda; ^3^Department of Biosystems Engineering, Faculty of Environmental Engineering and Mechanical Engineering, Poznan University of Life Sciences, Poznan, Poland; ^4^Department of Pharmaceutical Chemistry, College of Pharmacy, Taif University, Taif, Saudi Arabia; ^5^Faculty of Agriculture and Animal Sciences, Busitema University Arapai Campus, Soroti, Uganda; ^6^Faculty of Biomedical Sciences, Kampala International University Western Campus, Bushenyi, Uganda; ^7^Department of Clinical Pharmacy and Pharmacy Practice, School of Pharmacy, Kampala International University Western Campus, Bushenyi, Uganda; ^8^Department of Medical Microbiology and Immunology, Faculty of Medicine, Assiut University, Assiut, Egypt; ^9^Department of Basic Medical Sciences, Michael Chilufya Sata School of Medicine, Copperbelt University, Ndola, Zambia; ^10^Department of Pharmacology and Therapeutics, Faculty of Veterinary Medicine, Damanhour University, Damanhour, Egypt; ^11^National Research Center for Protozoan Diseases, Obihiro University of Agriculture and Veterinary Medicine, Obihiro, Japan; ^12^Zhejiang University-University of Edinburgh Institute, Zhejiang University School of Medicine, Zhejiang University, Haining, China

**Keywords:** bee venom, complementary medicine and alternative medicine, SARS-CoV-2 (2019-nCoV), pharmokinetics of bee poison, COVID-19 and complementary medicine, bee venom in clinical trials

## Abstract

Severe acute respiratory syndrome coronavirus 2 (SARS-CoV-2) is characterized by severe cytokine storm syndrome following inflammation. SARS-CoV-2 directly interacts with angiotensin-converting enzyme 2 (ACE-2) receptors in the human body. Complementary therapies that impact on expression of IgE and IgG antibodies, including administration of bee venom (BV), have efficacy in the management of arthritis, and Parkinson's disease. A recent epidemiological study in China showed that local beekeepers have a level of immunity against SARS-CoV-2 with and without previous exposure to virus. BV anti-inflammatory properties are associated with melittin and phospholipase A2 (PLA2), both of which show activity against enveloped and non-enveloped viruses, including H1N1 and HIV, with activity mediated through antagonist activity against interleukin-6 (IL-6), IL-8, interferon-γ (IFN-γ), and tumor necrosis factor-α (TNF-α). Melittin is associated with the underexpression of proinflammatory cytokines, including nuclear factor-kappa B (NF-κB), extracellular signal-regulated kinases (ERK1/2), and protein kinase Akt. BV therapy also involves group III secretory phospholipase A_2_ in the management of respiratory and neurological diseases. BV activation of the cellular and humoral immune systems should be explored for the application of complementary medicine for the management of SARS-CoV-2 infections. BV “vaccination” is used to immunize against cytomegalovirus and can suppress metastases through the PLA2 and phosphatidylinositol-(3,4)-bisphosphate pathways. That BV shows efficacy for HIV and H1NI offers opportunity as a candidate for complementary therapy for protection against SARS-CoV-2.

## Introduction

Severe acute respiratory syndrome coronavirus 2 (SARS-CoV-2) is the causal agent of coronavirus disease 2019 (COVID-19), a respiratory infection that emerged in Wuhan province of China in late 2019 (1, 2), becoming a global pandemic in 2020. By April 1, 2020, global mortality rates were reaching 5% (3). Within weeks, global mortality rates increased to 6.7% (5% for the African region, 4.4% for the Americas, 5% in the Eastern Mediterranean region, 4.4% for Southeast Asia, 8.9% for the European region, 4.4% in the Western Pacific region) (4). The public health challenges imposed by COVID-19 are immense, including management of the high number of asymptomatic cases (5). The disease has exacerbated existing socioeconomic disparities, especially in vulnerable communities in developing countries, including Africa, that have disproportionately been affected by the consequences of extreme preventative measures (6).

Severe SARS-CoV-2 infections are characterized by cytokine storm syndrome, hyperinflammation, and multiorgan failure (2, 7). Host cells are infected through the angiotensin-converting enzyme 2 (ACE-2) receptor (8, 9), associated with both innate and acquired immunity (10). ACE2 is considered to enhance viral replication and potentiate host cell invasion (10) and is a major component of the renin-angiotensin-aldosterone system (RAAS), interacting with enzymes of the CVS to cascade cardiovascular disease (11, 12). ACE2 may be the reason SARS-CoV-2 patients require pharmacological thrombosis prophylaxis (13, 14); the pathogenesis of SARS-CoV-2 involves viral binding to epithelial cells and local propagation with minimal innate immune response (15). The second stage of infection exhibits increased viral propagation, an active immune response, viral spread to the lower respiratory system, and may include cardiovascular and digestive systems (16). The third stage involves hypoxia, infiltration of the entire respiratory system, and finally acute respiratory distress syndrome (ARDS), which is potentially fatal (15). SARS-CoV-2 is associated with coagulopathies, thrombotic events (17) and lymphocyte exhaustion (18).

At present, there is no globally accepted alternative medical treatment protocol against SARS-CoV-2, although administration of polyclonal antibodies shows some promise (19). The efficacy of chloroquine and its derivatives continue to be explored for prevention of COVID-19 (20, 21) as well as Famotidine, an antiulcer drug, administered at high dosage (10× normal) for 14 days for control of SARS-CoV-2 infection (7). Remdesivir, which has previously been used to manage the Middle East Respiratory Syndrome-Cronavirus (MERS-CoV) has been explored as a candidate drug against SARS-CoV-2 (22–24). Combinations of Lopinavir/ritonavir, commonly used to prevent HIV/AIDS are also under investigation for efficacy against SARS-CoV-2 (25, 26). Neutrophil extracellular traps (NETs), common in snakes, insects, arachnids and myriapods have also been considered for SARS-CoV-2 (27, 28). Bee venoms (BVs) can act as ACE2 inhibitors or angiotensin-receptor blockers (ARBs), although studies on BVs and SARS-CoV-2 are sparse. Snake venom is known to act through phospholipase A2 (PLA2) to produce arachidonic acid, which induces hypotension (29). In humans, hymenoptera venom lowered key parameters in the RAAS (30). A combination of BV and propolis has been associated with hypotension in laboratory animals through a reduction in serum angiotensin levels (31), demonstrating a close relationship between BV and the ACE2 pathway.

## Bee Venom Therapy

Bee venom (BV) therapy dates back to the era of Hippocrates, where it was deployed to alleviate joint pain and arthritis (32). In contemporary medicine, BV is deployed for treatment of multiple sclerosis (33), arthritis and Parkinson's disease (34). Activity is based on anaphylactic reaction benefits on metabolism and on organelles, especially those of the respiratory system (35). Allergens may offer benefits against COVID-19 (36, 37); BV can induce elevation of specific IgE and IgG antibodies (38) and leads to production of IgE antibodies, which can respond to a variety of antigens (39) (Table 1). Although IgE are responsible for allergic outbursts, they also offer host protective roles over a wide range of allergens (39). BV can act as an adjuvant when combined with Toll-like receptor (TLR) ligands (40) and modulate the immune system, enhance the differentiation of foxP3-expressing cells and increase circulating regulatory T cells (41, 42). BV triggers an increase in CD25, CD4+ T cells and foxP3 mRNA, resulting in a shift in the BV-specific IgG4/IgE ratio (43). BV regulates the immune response and phsiopathological changes (44) and supports clinical observations in Apitherapy, where beekeepers were shown to mount immunity against COVID-19 in Wuhan province, PR China (45).

**Table 1:** Bee venom enzymes and peptides and their functions in mammalian systems.

**References**	**Component**	**Compound**	**Properties/mode of action**	**% BV**	**Properties / mode of action for mammalian analog**
Dams and Briers (130) Wehbe et al. (103)	Enzyme	Hyaluronidases	Breakdown of hyaluronic acid to increase tissue permeability, accelerated distribution of toxins “spreading factor” Increases bioavailability of drugs, used in therapy of extravasations, management of complications associated with aesthetic injection of hyaluronic acid-based fillers	1–3	Ubiquitous in somatic tissues Six forms in humans (HYAL1-4, HYAL-P1, and PH-20) PH-20 has highest activity, highest in testicles and involved in fertilization process Breaks down tissue hyaluronic acid and chondroitin sulfate increasing tissue permeability e.g., of sperm during adhesion and penetration to cumulus oophorus
Boens et al. (131) Szulc and Bauer, (131, 132)	Enzyme	Acid phosphatases	Hydrolyzing monophosphate esters to release products associated with pain and inflammation, Potent releaser of histamine in human basophils, thus relevant in allergic process	1	In prostate, erythrocytes, macrophages, platelets, bones, spleen, lungs, testes Hydrolyzes phosphate Enzyme dysregulation is associated with pathophysiological conditions e.g., prostatic acid phosphatase (cancer of prostate); tartrate-resistant acid phosphatase (abnormal bone resorption in osteoporosis) Released by platelets during clotting. Binding to α2-macroglobulin leads to a reduction in its activity
Murakami et al. (133) Stahelin (134)	Enzyme	Phospholipase A2 (PLA_2_)	Most lethal enzyme in BV Formation of melittin-PLA_2_ complex referred to as the bee hemolytic factor that cleaves cellular membrane phospholipids and cellular lysis Potent allergen Trypanocidal, antibacterial, neuronal protection, anti-tumor properties. Hepato-protective in acetaminophen-induced liver damage	10–12	Ubiquitous in many cells and tissues (pancreas, spleen, liver, intestines, spleen, lung, heart, testis, brain, macrophages, inflamed tissues, and inflammatory cells). Involved in inflammation: generation of precursors of eicosanoids (prostaglandins, leukotrienes), platelet-activating, factor; cell activation via a specific receptor; digestion and metabolism of dietary phospholipids; host defense and signal transduction, exocytosis, antimicrobial activity, anticoagulation, ischemia, brain development Overproduction of lipid mediators associated with PLA_2_ activity can cause inflammation and tissue disorders PLA_2_ is expressed in alveolar macrophages during inflammation to clear lung exudates, and by cytokine induction and airway dysfunction
Connolly et al. (135) Lima and Brochetto-Braga (91)	Enzyme	Phosphomonesterase	Acid phosphatase with similar properties	1	Found in accessory reproductive organs (prostate and seminal vesicles) and in other parts of the genital tract (testis, vas deferens, epididymis) Hydrolyses choline-O-phosphate Involved in calcium metabolism during blood clotting Alkaline phosphomonesterases involved in wound healing Activity increased in kidney from dioxydin accumulation.
Brás et al. (136)	Enzyme	α-glucosidase	Associated with honey production by bees	0.6	Four human forms in digestive system [salivary and pancreatic α-amylases (endohydrolase); α-maltotriose (oligoglucans); α-maltase-glucoamylase and α-sucrose-isomaltase (exohydrolases)] Essential for digestion of starch to glucose Facilitates glucose absorption especially by enterocytes Involved in metabolic disorders such as type 2 diabetes and obesity due to hyperglycemia Application for anti-diabetic agents
Holtsberg et al. (137) Karamitros and Konrad (137, 138)	Enzyme	Lysophospholipase	Increases PLA2 activity. PLA2 degrades phospholipids into lisophospholipids that are cleaved by the lysophospholipases into glycerophosphocoline and anionic fatty acids	1	Found in eosinophils, pancreas, brain, liver, lactating mammary glands, and most (if not all) cells Breaks down phosphatidylcholine to glycerophosphate-choline to release choline. Hydrolyses lysophospholipids and attenuates lysophosphatidic acid-mediated signal transduction in nervous tissues Pancreatic form is involved in digestion Eosinophilic form is involved in immunologic function Those with an N-terminal L-Asparaginase domain have role in amino acid metabolism useful in medical and therapeutic applications e.g., antileukemic protein drug
Soliman et al. (57, 139) Pucca et al., (57, 139)	Peptide	Melittin	Most toxic component Attacks lipid membranes causing cell lysis, haemolysis, cytotoxicity, and biodegradation Forms melittin-PLA2 complex that causes cellular injury Induces mild allergic but severe pain reactions In cancer therapy due to its cytotoxic activity on cancer cells Control of excessive immune responses Anti-inflammatory, antimicrobial, antiviral, fungicidal, and anti-cancer properties	40–60	
Pucca et al. (57, 140) Issa et al., (57, 140)	Peptide	Apamin	Inhibits Ca^2+^-dependent K^+^ channels (blocks potassium permeabilities) facilitating the crossing of the blood brain barrier Causes neurotoxic effects such as intense local pain, hyperactivity, seizures, tonic-clonic convulsions, jerks, spasms Potential treatment for neurological disabilities such as learning deficit disorder, Parkinson's disease by activating of inhibitory muscarinic receptors of motor nerve terminals	1–3	Tissues with acceptor receptors for apamin in lower mammals: rat brain, rat neuroblastoma, rat and guinea pig liver, guinea pig colon, synaptosomes, rat myotubes
Moreno and Giralt (85).	Peptide	Mast cell degranulating peptide	Inflammatory and anti-inflammatory properties: inflammation/allergy: at low concentration it induces massive release of histamine, serotonin and vasoactive amines from mast cells -anti-inflammatory/ anti-allergic: in high quantity it inhibits mast cell degranulation by inhibiting histamine Can cause hyperexcitability in mammalian neurons (convulsant) Potential to induce allergy and inflammation by inducing secretion of mast cells, basophils, and leukocytes is of value in designing therapeutic compounds	2–3	
Seo et al. (77) Cherniack and Govorus (76) Gu et al. (78)	Peptide	Adolapin	Inhibits cyclooxygenase activity and blocks prostaglandin synthetase system leading to antipyretic, anti-inflammatory and anti-nociceptive/analgesic cascades Inhibits lipoxygenase from human platelets Elevates the c-GMP level in rat spleen and brain and inhibits phospholipase A2, c-AMP in rats' spleen Utilized in bee venom acupuncture to successfully manage musculoskeletal diseases (lumbar disc disease, osteoarthritis of the knee, rheumatoid arthritis, adhesive capsulitis, and lateral epicondylitis)	1	
Elieh et al. (141) Rady et al. (79, 141)	Amines	Histamine	Mediators of allergic and inflammatory reactions. Can cause anaphylactic responses, sometimes leading to cerebral or myocardial ischemia Can cause pain by affecting neurons or through release of pain-inducing chemicals	1.5	Mediators of local allergic and inflammatory reactions Physiological modulators in tissues/organs of gut, nervous system, blood etc. Act as neurotransmitters Role in fight-or-flight response (adrenaline/noradrenaline)
		Dopamine		0.1–1	
		Noradrenaline		0.1–0.7	
		Serotonin		0.1–0.5	
		Adrenaline		0.1–1	
	Pheromones	Iso-pentyl acetate; n-butyl acetate; iso-pentanol; n-hexyl acetate; n-octyl acetate; 2-nonanol; n-decyl acetate; benzyl acetate; benzyl alcohol; (2)-11-eicosen-1-ol	In bees: Cause alarm and loyal pheromones after evaporation from the surface of the sting alert, attract other bees to the marked target Affect physiological changes through the autonomous nervous system, inflammatory signaling, immune system changes and/or behavioral change.	4–8	Control of innate social behaviors and regulation of hormone levels.

The *bv*PLA2 can trigger mast cell maturation (46), is important in cell signaling and for production of key lipids and may act as a receptor ligand (47). PLA2 can inhibit the flow of inflammatory cells to targets (48). BVs may lead to lasting induced tolerance to related allergens (49), as a function of reducing IgG4 and activating IL-10, modulating the immune system and inducing deviation from TH2 to TH1(50–52). Melittin (APi M 1) can be used to develop mimotopes (49). APi M 10 (icarapin), a BV component, activates effector cells of honey bee venom allergic patients (53). Since IgE possesses an epitope for APi M 10, this may offer opportunity for adjuvant development. BV antigens can be used as adjuvants in the treatment of pain (54) and the action of melittin on cell membrane pore formation (54, 55), leading to apoptosis serves to strengthen adjuvant properties. BV also has antiviral properties (56). BV can desensitize mast cells and basophils (57) and suppress innate lymphoid cells. BV materials can inhibit proteinsynthesis, induce angiogenesis (58) and activate caspase-3-8-9 (59) (Table 1).

## Conditions That Allow Bee Venom Use Despite Its Toxicity

Bee venom is cytotoxic at high doses, however, non-cytotoxic concentrations of BV range from 1 to 3 μg/ml, show significant therapeutic potential (60). Low doses, controlled concentrations, and diluted BVs trigger a range of anti-inflammatory responses (61, 62), and have been deployed for management of diabetes, rheumatoid arthritis (RA), heart disease, obesity, asthma, skin diseases, and central nervous system-associated diseases, such as Alzheimer's disease, Parkinson's disease, and sclerosis (61–64). At low doses, BV can suppress inflammatory cytokines such as interleukin-6 (IL-6), IL-8, interferon-γ (IFN-γ), and tumor necrosis factor-α (TNF-α). A decrease in the signaling pathways responsible for the activation of inflammatory cytokines, such as nuclear factor-kappa B (NF-κB), extracellular signal-regulated kinases (ERK1/2) and protein kinase Akt, and porphyromonas gingivalis lipopolysaccharide (PgLPS)-treated human keratinocytes has been associated with treatments involving BV (65).

BV has been used as an anti-inflammatory agent by combining compounds in BV, i.e., secretory phospholipase A2, with phosphatidylinositol-(3,4)-bisphosphate or cells, mainly dendritic cells (DCs), or combining BV with DCs (66). Conjugation of hormone receptors and gene therapy transporters to BV peptides as a useful novel targeted therapy to positively modulate immune responses has been applied in anticancer and anti-inflammatory therapy (67).

BV immune reactions are toxic at high doses but when controlled or diluted (controlled concentrations) these immune reactions can serve as immune modulators. Controlled allergic immunity can be advantageous for host defense against antigens and pathogens including RNA viruses. BV can stimulate type 2 immune responses, type 2 immunity is initiated by T-cell (T-helper type 2) and immunoglobulin (Ig) antibodies (IgE and IgG1) and the action of the innate immune system, such as the epithelium and white blood cells and serves as a barrier defenses to eliminate antigens (68). BV group III sPLA2 shows *in vitro* and *in vivo* effects on the immune system. Modulated immune reactions from BV can alleviate immunological illnesses such as rheumatoid arthritis, inflammatory illnesses, asthma, and Parkinson's disease (69). The innate immune system induces a defensive immune response against BV antigens through pattern-recognition receptors (PRRs), including Toll-like receptors found on pathogen-associated molecular patterns (PAMPs) (70). BV in therapeutic disease, is anti-inflammatory (44) decreasing numbers of infiltrated inflammatory cells, and the expression of tumor necrosis factor (TNF)-α, interleukin (IL)-1β, inhibition Toll-like receptor (TLR)2 and CD14. BVs also suppress the binding potential of nuclear factor-κB (NF-κB) and activator protein (AP)-1 (71). Human IL-1 receptor (anakinra) also shows anti-inflammatory activity (72), however information linking this receptor and Bee venom remain sparse.

Bee venom phospholipase 2 (*bv*PLA2) is the main allergen in BV and stimulates the innate immune system by binding to pattern-recognition receptors (PRRs), e.g., Toll-like receptors that recognize pathogen-associated molecular patterns (PAMPs), triggering a type 2 immune response. *bv*PLA2 induces T-helper cell-type reactions and group 2 innate lymphoid cells (ILC2s) facilitated through the enzyme-aided cleavage of membrane phospholipids and secretion of IL-33. *bv*PLA2 also induces the production of IgE shown to be protective in mice from future allergic/immunologic reactions [in the case of a lethal dose of BV (70)]. PLA2 plays a vital role in host defense in Th2 differentiation, ILC2 activation, immunoglobulin production, membrane remodeling, and anti-inflammatory reactions (44, 70).

**Figure 1:**
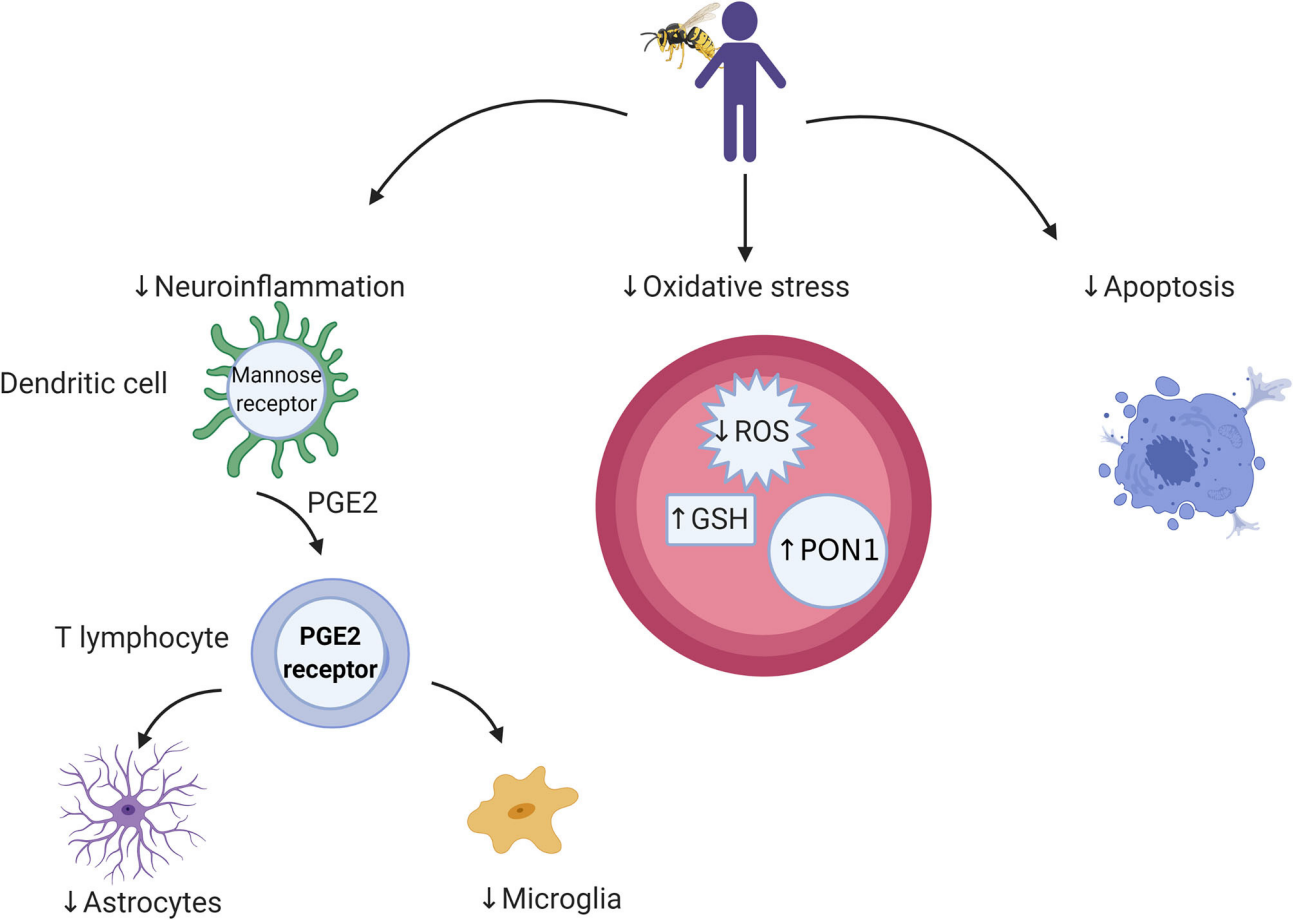
Cellular and microbial targets relevant to bee venom components and targets for future research. Bee venom acts through dendritic cells to stimulate the immune system through which it activates cellular immunity. Its antioxidant activity is associated with a reduction in reactive oxygen species (ROS) and elevation in antioxidant enzymes (e.g., GSH and PON1), which leads to protection against cell death.

BV shows positive immune-modulating roles; reducing the progression of tumors and activating the immune system by combining *bv*PLA2 with phosphatidylinositol-(3,4)-bisphosphate or cells, mainly dendritic cells (DC) (66). DCs prepared with BV *in vivo* have both anticancer and antiviral properties. DCs combined with antigens from a tumor or virus produce major histocompatibility complex (MHC) class I and II peptides epitopes to CD8 and CD4 T lymphocytes (Figure 1).

PLA_2_ (bvPLA_2_-H34Q) is membrane-binding and *in vivo* combines antigens with the human DC cell membrane, causing stimulation of CD8 T cells and antiviral and antitumor vaccines (DC vaccine) can be obtained from BV using DCs. These cell-based antiviral/antitumor vaccines are used during immunization against viruses including cytomegalovirus and for tumor suppression (73, 74). BV is a known adjuvant-potentiated antimicrobial and antitumor vaccine. Melittin, bvPLA2 and phosphatidylinositol-(3,4)-bisphosphate are effective adjuvants for anti-leishmania, anti-tumor and anti-cytomegalovirus vaccines (73–75). Conjugation of BV peptides with hormone receptors and gene therapy offer to positively modulate immune responses applied offer targeted anticancer and anti-inflammatory therapies (67).

BV can be used as an analgesic at controlled dose concentrations; inhibiting cyclooxygenase activity and blocking the prostaglandin synthetase system, leading to antipyretic, anti-inflammatory, and anti-nociceptive/analgesic cascades (76–78). In diluted form BV can induce anti-nociceptive effects via the α-adrenergic receptor (activation of the spinal α-adrenergic receptor) (61, 62). Conjugation of BV peptides to protein receptors such as hormones and genes transporting the peptides provides an innovative BV controlled anti-inflammatory, anti-nociceptive, and immune-modulatory therapy (67).

## Pharmacodynamics of Bee Venom Constituents

Bee venom (BV) contains enzymes [phospholipase A2 (PLA_2_), phospholipase B, hyaluronidases, acid phosphatases, acid phosphomonesterases, α-D-glucosidases, and lysophospholipases]; peptides [lytic peptide melittin, apamin, mastocyte (mast cell) degranulating peptide, secapine, pamine, minimine, procamine A, B, protease inhibitor, tertiapine, cardiopep, and adolapin] (30–33); and amino acids include g-aminobutyric acid and a-amino acids. Non-peptide components include amines (dopamine, histamine, norepinephrine, neurotransmitters), carbohydrates (glucose, fructose), pheromones (iso-pentyl acetate; n-butyl acetate; iso-pentanol; n-hexyl acetate; n-octyl acetate; 2-nonanol; n-decyl acetate; benzyl acetate; benzyl alcohol; and (2)-11-eicosen-1-ol) (79, 80) (Table 1).

BV has been shown to have anti-inflammatory, antinociceptive, antioxidant, and anti-apoptotic properties and has been shown to alter gene expression and fibrosis (81–84). Side effects include proinflammation [higher doses of PLA2, mast cell degranulating peptides, hemolytic compounds (melittin)], allergic reactions to protease inhibitors and peptides, anaphylactic responses and death (76).

Multiple protein allergens in bee venom are responsible for the allergic response (85). Allergic reactions can take place in the respiratory system, gastrointestinal system, cardiovascular system, skin and stings and can result in severe anaphylactic shock, sometimes leading to cerebral or myocardial ischemia (86, 87). A non-immune-mediated mechanism of allergy to BV involves the production of bradykinin (BK) mediators, leading to anaphylaxis (88) from melittin activation of PLA2 (mimicking BKs).

## Biological Variability of Bee Venom Composition Among Bee Variants for Biotoxin Administration in Complementary Medicine

Bees and wasps belong to the insect order Hymenoptera (89, 90). In bees, venom production is highest for queen bees on emergence. Hymenoptera venom causes toxic or allergic reactions mostly caused by biochemical compounds associated with local inflammatory action (91, 92). Stings defend the colony in all insects of the order Hymenoptera (93, 94). Melittin is the most prominent compound responsible for these allergic reactions (95, 96); although a combination of mastocytes with IgE invokes activity of leucotrienes, histamines and platelet activating factors during allergic reactions (93, 94, 97).

Hymenoptera venoms contain dopamine, adrenaline, hyaluronidase, noradrenaline, serotonin, histamine, phospholipases A and B (85) but only BV contains mast cell-degranulating peptide, melittin and apamin (57). Different bee species bees; *Apis mellifera mellifera and Apis mellifera ligustica* (in Europe) *and Apis mellifera scutellate (in Africa)* are responsible for human envenoming (57). The median lethal dose of BV ranges from 2.8 to 3.5 mg/kg body weight, and on average, 140–150 μg of BV is injected in a stinging episode (57). The chances of death from only a few bee stings is minimal in non-allergic persons (98) and the severity of the envenomation is duly influenced by the body weight, age and immune status of the victim (99, 100). Sting number and any previous sensitization to BV affect envenoming severity (99, 100).

BV is a clear, odorless, colorless watery liquid with a pH of 4.5–5.5 with a bitter taste and in some cases an ornamental pungent smell (101, 102). BV composition is affected by extraction methods due to its volatility (101). *Apis mellifera* venom is arguably the best characterized venom in Hymenoptera (103). Venom from all *Apis* species is similar in composition and quality. *A. florea*, a honey bee is smallest in its family, while *A. dorsata* is the largest (101). *Apis cerana* venom is twice as tocic as that of *Apis mellifera* (104). Differences in the composition of venom gland and venom sac secretion and the concentration of lipids, proteins, activity of acid phosphatase and hexokinase have been observed in the venom glands of *A. dorsata* > *A. cerana* > *A. mellifera* > *A. florea*. Lipid, protein, carbohydrate and alkaline phosphatase compositions have been found to be in the order of *A. cerana* > *A. mellifera* > *A. florea*. Glycogen was absent in both the venom gland and venom sac of Apis species (101).

Variability in bee venom composition is related to species, age, geographic localization and social condition (96). Young worker bees have lower concentrations of melittin and histamine and higher concentrations of apamin than older worker bees (57). Queen bees have low concentrations of melittin and apamin and high concentrations of histamine (57). APi M reaches its peak when the bee is ~28 days old and declines with age (105). Levels of PLA2 reach a maximum at around day 10 of hatching (101). African bees release small quantities of venom in stinging episodes, with high concentrations of PLA2 and reduced concentrations of melittin and hyaluronidase (57). Seasonal variations in the composition of the BV have been reported (106); for example, during winter, APi M production increases but decreases during summer (107, 108).

## Current Therapeutic Advances of Bee Venom

### Antiviral and Antibacterial Properties

**Figure 2:**
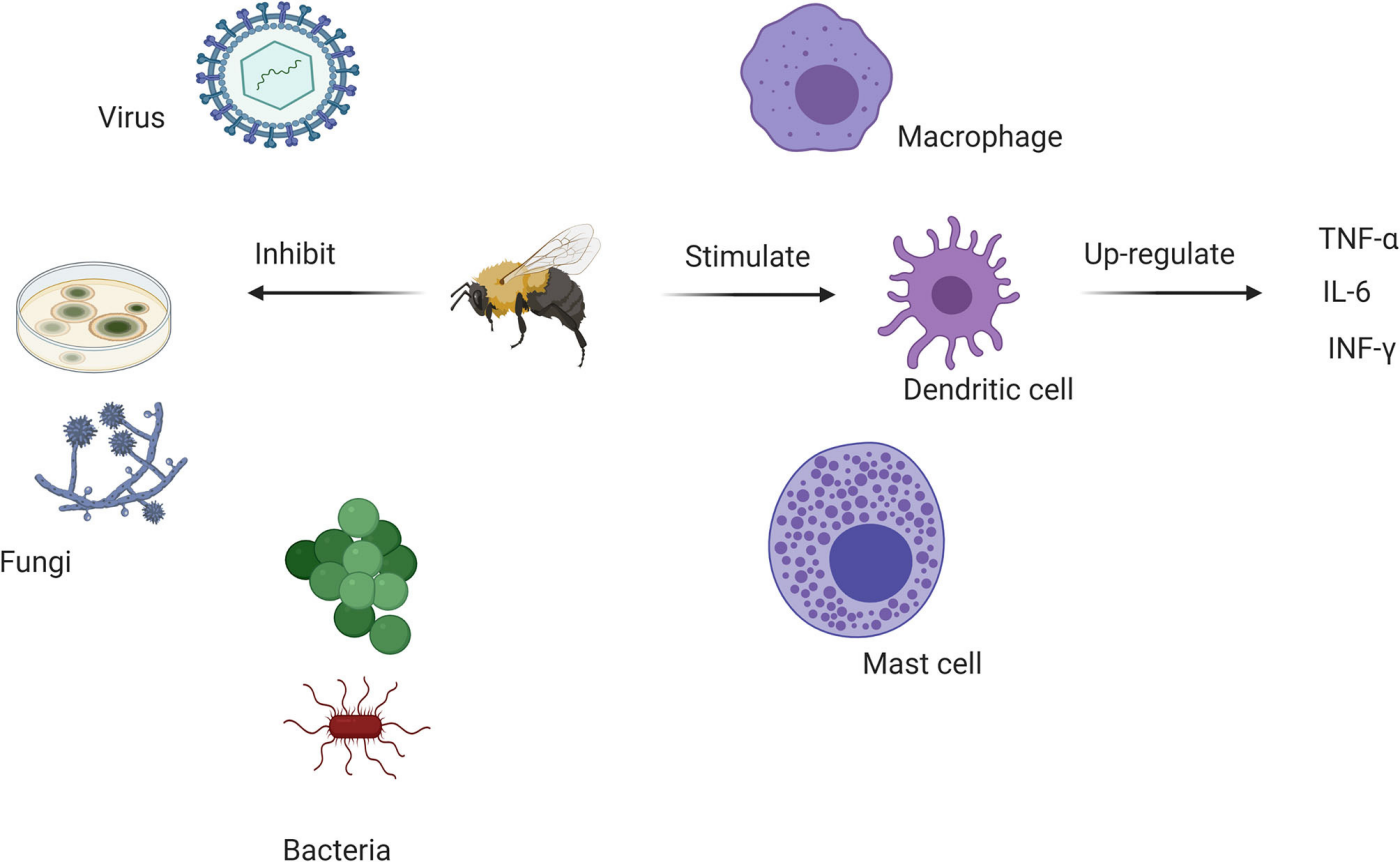
Antimicrobial and immunomodulatory actions of various bee venom components. BV inhibits bacterial, antifungal and viral growth while stimulating dendritic cell activity through major anti-inflammatory cytokines. This offers a rationale for its use in complementary medicine to control the SARS-CoV-2 pandemic.

Melittin and PLA2 exhibit antimicrobial activities and have been used as complementary antibacterial agents (103); inducing pore formation and destruction of bacteria (109). APi M shows antiviral properties against some enveloped viruses and non-enveloped viruses *in vitro* (110). Protection has been observed in mice following exposure to influenza A H1N1 virus but BV can also interact directly the viral surface (110) (Figure 2).

### Management of Cancer

BV has been explored in cancer (111, 112); melittin is considered cytolytic but non-specific. Melittin can break down the membrane lipid bilayer and exhibits toxicity when injected intravenously (113). APi M has the ability to suppress tumor growth in breast, liver, prostate, and lung cancer cells (111, 112). *In vitro* and *in vivo* studies show that melittin can suppress growth of cancerous cells by inhibiting NF-κB signaling and activating caspase 3 and 9 pathways. Inhibition of hepatocellular carcinoma cell motility was observed *in vitro* and *in vivo* by suppression of Rac1-dependent pathways (114).

### Anti-inflammatory Potential

Low doses of BV trigger a range of anti-inflammatory responses that have been explored in diabetes, rheumatoid arthritis (RA), heart diseases, obesity, asthma, skin diseases, and central nervous system-associated diseases (Alzheimer's disease, Parkinson's disease, and amyotrophic lateral sclerosis) (63, 64). BV suppresses inflammatory cytokines, including interleukin-6 (IL-6), IL-8, interferon-γ (IFN-γ), and tumor necrosis factor-α (TNF-α). A decrease in the signaling pathways responsible for the activation of inflammatory cytokines, nuclear factor-kappa B (NF-κB), extracellular signal-regulated kinases (ERK1/2) and protein kinase Akt, and Porphyromonas gingivalis lipopolysaccharide (PgLPS)-treated human keratinocytes are associated with melittin treatment(65) (Figure 2).

## Host Responses to Bee Venom

BV therapy can alleviate immune-related illnesses. Group III secretory phospholipase A_2_ from BV (BV group III sPLA_2_) shows *in vitro* and *in vivo* activity on the immune system and has been used to manage asthma, Parkinson's disease, and drug-induced organ inflammation (69). BV immune reactions can be dangerous when highly elevated, but when controlled, allergic immunity can be advantageous in host defense to stimulate type 2 immune responses. Type 2 immunity is mainly based on barrier defenses, and these responses are initiated by T helper type 2 (T_H_2) cells, immunoglobulins E and G1 (IgE and IgG1) antibodies, and other components of the innate immune system (epithelial barriers, innate lymphoid cells-ILCs, eosinophils, mast cells, basophils, and activated macrophages) (68). The innate immune system senses components of venom, inducing a defensive immune response against antigens through pattern-recognition receptors (PRRs), e.g., Toll-like receptors found on pathogen-associated molecular patterns (PAMPs) (70). BV anti-inflammatory properties (44) may inhibit the activity of inflammatory antigens, reduce the number of infiltrated inflammatory cells, and inhibit the expression of (TNF)-α, IL-1β, Toll-like receptor (TLR)2 and CD14 expression, suppressing the binding activity of nuclear factor-κB (NF-κB) and activator protein (AP)-1 (71). The main Bet V 1 allergen, PLA2, stimulates the innate immune system, binding to PRRs, e.g., Toll-like receptors that recognize PAMPs, triggering a type 2 immune response in mice. PLA2 in BV induces T helper type 2 (Th2) cell-type reactions and group 2 innate lymphoid cell (ILC2) activation via the enzymatic cleavage of membrane phospholipids and secretion of IL-33. PLA2 induces the production of IgE, protecting mice from future allergic/immunologic reactions in the case of a lethal dose of BV (70); PLA2 plays a critical role in host defense by improving Th2 differentiation, ILC2 activation, immunoglobulin production, membrane remodeling, and anti-inflammatory reactions (44, 70).

## Bee Venom Vaccines

BV can suppress the progression of tumors and activate the immune system by combining secretory phospholipase A2 in BV with compounds including phosphatidylinositol-(3,4)-bisphosphate or dendritic cells (DCs) (66). DCs treated with BV *in vivo* show anticancer and antiviral properties. DCs combined with antigens from a tumor or virus can produce major histocompatibility complex (MHC) class I and class II peptide epitopes to CD8 and CD4 T lymphocytes, leading to a series of immune reactions in response to the antigens. BV phospholipase A_2_ (bvPLA_2_-H34Q) is membrane-binding and links antigens within the cell membrane of human DCs *in vivo*. This induces recognition by and activation of CD8 T cells with the implication that antiviral and antitumor vaccines may be derived from BV (DC vaccine). Vaccines from BV and DCs (cell-based antiviral/antitumor vaccines) are used for immunization against viruses such as cytomegalovirus and for suppression of tumors (73, 74). BV can be used as a potent adjuvant-potentiated antimicrobial and antitumor vaccine and shows potential in vaccines where melittin. sPLA2 and phosphatidylinositol-(3,4)-bisphosphate are effective adjuvants (anti-leishmania, antitumor and anti-cytomegalovirus vaccines) (73–75).

A leading adjuvant of SARS-CoV-2 therapies currently being promoted is aluminum hydroxide due to its slow release and increased interaction with antigen presenting cells (115). Bee venom offers a candidate for control SARS-CoV-2 infections and could offer advantages against COVID-19. PLA_2_ has been associated with a level of success against SARS-CoV-2 infections (116, 117). Conjugation of BV peptides could offer a new approach in the development of the BV vaccine.

## Potential Relationship Between Bee Venom Proteins and COVID-19 Proteins

SARS-CoV-2 belongs to the β-coronavirus genus. SARS-CoV-2 has four obvious structural proteins: membrane, spike, nucleocapsid proteins, and envelope. The structural integrity of the SARS-CoV-2 virus is maintained by structural proteins and forms a protective coat around its RNA. The coronavirus membrane contains 3 or 4 viral proteins (118, 119), the membrane glycoprotein is the most abundant structural protein and spans the membrane bilayer three times, with a long COOH terminus inside the virion and a short NH2-terminal domain outside the virus (120). The SARS-CoV-2 genome encodes several reading frames (ORFs); ORF1a/b codes for 16 non-structural proteins and translates two polyproteins (pp1a and pp1ab) accounting for up to 2/3 of the viral RNA. The remaining ORFs code for structural proteins (spike glycoprotein, matrix protein, nucleocapsid protein, and small envelope protein) (118, 119). SARS-CoV-2 has accessory proteins that interfere with the innate immune response of the host (118).

The spike protein is usually a Type I membrane glycoprotein and constitutes the peplomers, known for involvement in antibody interaction. The membrane plays a significant role in the intracellular formation of virus particles independent of the viral spike. Coronaviruses grow and produce spikeless forms in the presence of tunicamycin, thereby resulting in the production of non-infectious virions that contain membranes but without spikes (118).

Melittin can puncture the protective membrane envelopes surrounding viruses, including human immunodeficiency virus (HIV) (119). Many viruses, including SARS-CoV-2, rely on their protective envelope and may be vulnerable in melittin-guided bee venom therapy (Table 1).

The phospholipase A2 components of bee venom have the potential for antiviral activities (121). Melittin-loaded nanoparticles delivered a significant amount of melittin intravenously, targeting and killing precancerous lesions in K14-HPV16 mice with squamous dysplasia and carcinoma containing human papillomavirus (HPV) transgenic elements (E6 and E7 oncogenes) (122, 123).

In Hubei Province, the epicenter of the SARS-CoV-2 outbreak in China, the local beekeeper association surveyed 5,115 beekeepers between 23rd February and 8th March (including 723 in Wuhan) and showed that none developed any symptoms observed for COVID-19 patients (124). Five apitherapists in Wuhan and 121 of their patients who had received apitherapy between October and December 2019 were interviewed; two apitherapists were exposed to suspected and/or confirmed COVID-19 victims without protection. None of the apitherapists developed symptoms associated with SARS-CoV-2 and none of their 121 patients contracted COVID 19, despite 3 having been exposed to SARS-CoV-2-infected relatives (124).

Apitherapy employs honeybees and their products (BV, honey, royal jelly, pollen, propolis, beeswax). BV therapy uses venom to modulate the body's immune system and improve/facilitate healing and includes either the use of live bee stings or injectable venom for the management of arthritis, rheumatoid arthritis, multiple sclerosis (MS), lupus, sciatica, low back pain, and tennis elbow (125, 126). Hymenopteran products are potent accelerators of wound healing (127). Insect venoms are less complex and less variable in composition and physiological activity compared to snake venoms (125, 126). BV can be administered to induce allergic immune responses, stimulating the innate immune system of the host (68), due to the presence of allergens that promote the type 2 immune responses (44, 68–71). BV antiviral and antitumor action when BV secretory phospholipase A2 is mixed with other compounds, such as phosphatidylinositol-(3,4)-bisphosphate or dendritic cells, and/or bee proteins, such as melittin, is advantageous (66) and employed in the production of cell-based antiviral/antitumor vaccines (73–75). The immunological properties of BV are also found in natural products that mimic bee venom (127, 128), and further studies regarding the role of bee venom as a potential candidate for use in complementary medicine for the management of viruses such as SARS-CoV-2 could consider other natural products that mimick BV activity.

## Future Research on Bee Venom

The development of adjuvant therapies (using APi M and PLA2) to use against SARS-CoV-2 infections offers a unique approach to viral therapy. Bee venom vaccine development using DCs using APi M and *bv*PLA2 offers a new opportunity for complementary medicine interventions against SARS-CoV-2 infections. Studies to examine cellular signaling between BV proteins, Janus Kinase (JAK) and activator of transcription (JAK-STAT) would help strengthen its adoption in complementary medicine against SARS-CoV-2. Inhibitors of JAK are associated with improved prognosis in COVID-19 patients (72, 129) but studies are needed to elucidate the cellular mechanisms. Synergistic activity through combinations in alternative and complementary medicine would help combat side effects associated with current monotherapies for the management of SARS-CoV-2 infections. SARS-CoV-2 is a novel virus and novel therapies may be needed to support management over time and may be of value in supporting the immune response in patients suffering from so called long-COVID.

## Conclusion

SARS-CoV-2 effects on the ACE2 receptors have been associated with severe inflammatory activity and a poor prognosis, depending on the co-morbities of the patient and other associated risk factors. Even if patient recover from initial infection, they may be faced with a long and complicated convalescence and/ or so called, long-COVID. It is unlikey that there will be a magic bullet therapy for COVID-19 soon, and complimentary therapies should be explored that compliment conventional therapy and support healthy recovery. BV melittin and phospholipase A2 activity have strong anti-inflammatory action and could be deployed to support recovery. That BV has successfully been used to manage neurological and immunological diseases strengthens the case for exploration of its use in complimentary medicine for SARS-CoV-2 infections. BV is a potential adjuvant against COVID-19 which should be added to the list of major therapies.

## Author Contributions

All authors listed have made a substantial, direct and intellectual contribution to the work, and approved it for publication.

## Conflict of Interest

The authors declare that the research was conducted in the absence of any commercial or financial relationships that could be construed as a potential conflict of interest.

## References

[1] Ji Y Ma Z Peppelenbosch MP Pan Q Potential association between COVID-19 mortality and health-care resource availability. Lancet GlobHealth. (2020) 8:e480 10.1016/S2214-109X(20)30068-1 PMC7128131 32109372

[2] Mehta P McAuley DF Brown M Sanchez E Tattersall RS Manson JJ. COVID-19: consider cytokine storm syndromes and immunosuppression. Lancet. (2020) 395:1033–4. 10.1016/S0140-6736(20)30628-0 32192578 PMC7270045

[3] WHO Coronavirus disease 2019. (COVID-19) Situation Report-72 HIGHLIGHTS. (2020). Available online at: https://www.who.int/docs/default-source/coronaviruse/situation-reports/20200401-sitrep-72-covid-19.pdf?sfvrsn=3dd8971b_2 (accessed September 15, 2020).

[4] WHO Coronavirus disease 2019. (COVID-19) Situation Report – 88. (2020). Available online at: https://www.who.int/docs/default-source/coronaviruse/situation-reports/20200417-sitrep-88-covid-191b6cccd94f8b4f219377bff55719a6ed.pdf?sfvrsn=ebe78315_6 (accessed September 15, 2020).

[5] Mizumoto K Kagaya K Zarebski A Chowell G. Estimating the asymptomatic proportion of coronavirus disease 2019. (COVID-19) cases on board the Diamond Princess cruise ship, Yokohama, Japan, 2020. Eurosurveillance. (2020) 25:1–5. 10.2807/1560-7917.ES.2020.25.10.2000180 32183930 PMC7078829

[6] Quaresima V Naldini MM Cirillo DM. The prospects for the SARS-CoV-2 pandemic in Africa. EMBO Mol Med. (2020) 12:e12488. 10.15252/emmm.202012488 32301279 PMC7235494

[7] Ghosh R Chatterjee S Dubey S Lavie CJ Famotidine against SARS-CoV2: a hope or hype? Mayo Clin Proc. (2020) 95:1797–9. 10.1016/j.mayocp.2020.05.027 32753153 PMC7275146

[8] Clerkin KJ Fried JA Raikhelkar J Sayer G Griffin JM Masoumi A COVID-19 and cardiovascular disease. Circulation. (2020) 141:1648–55. 10.1161/CIRCULATIONAHA.120.046941 32200663

[9] Zheng YY Ma YT Zhang JY Xie X. COVID-19 and the cardiovascular system. Nat Rev Cardiol. (2020) 17:259–60. 10.1038/s41569-020-0360-5 32139904 PMC7095524

[10] Li G He X Zhang L Ran Q Wang J Xiong A. Assessing ACE2 expression patterns in lung tissues in the pathogenesis of COVID-19. J Autoimmun. (2020) 112:102463. 10.1016/j.jaut.2020.102463 32303424 PMC7152872

[11] Oudit GY Crackower MA Backx PH Penninger JM. The role of ACE2 in cardiovascular physiology. Trends Cardiovasc Med. (2003) 13:93–101. 10.1016/S1050-1738(02)00233-5 12691672

[12] South AM Diz DCM. COVID-19, ACE2, and the cardiovascular consequences. Am J Physiol Circ Physiol. (2020) 318:H1084–90. 10.1152/ajpheart.00217.2020 32228252 PMC7191628

[13] Klok FA Kruip MJHA van der Meer NJM Arbous MS Gommers D Kant KM. Confirmation of the high cumulative incidence of thrombotic complications in critically ill ICU patients with COVID-19: an updated analysis. Thromb Res. (2020) 191:148–50. 10.1016/j.thromres.2020.04.041 32381264 PMC7192101

[14] Mehra MR Desai SS Kuy SR Henry TD Patel AN Cardiovascular disease, drug therapy, and mortality in COVID-19. N Engl J Med. (2020) 382:E102 10.1056/NEJMoa2007621 32356626 PMC7206931

[15] Mason RJ. Pathogenesis of COVID-19 from a cell biology perspective. Eur Respir J. (2020) 55:9–11. 10.1183/13993003.00607-2020 32269085 PMC7144260

[16] Rothan HA Byrareddy SN. The epidemiology and pathogenesis of coronavirus disease (COVID-19) outbreak. J Autoimmun. (2020) 109:102433. 10.1016/j.jaut.2020.102433 32113704 PMC7127067

[17] Zhang Y Xiao M Zhang S Xia P Cao W Jiang W. Coagulopathy and antiphospholipid antibodies in patients with Covid-19. N Engl J Med. (2020) 382:e38. 10.1056/NEJMc2007575 32268022 PMC7161262

[18] Cao X. COVID-19: immunopathology and its implications for therapy. Nat Rev Immunol. (2020) 20: 269–70. 10.1038/s41577-020-0308-3 32273594 PMC7143200

[19] Duan K Liu B Li C Zhang H Yu T Qu J. Effectiveness of convalescent plasma therapy in severe COVID-19 patients. Proc Natl Acad Sci USA. (2020) 117:9490–6. 10.1073/pnas.2007408117 32253318 PMC7196837

[20] Cortegiani A Ingoglia G Ippolito M Giarratano A Einav S. A systematic review on the efficacy and safety of chloroquine for the treatment of COVID-19. J Crit Care. (2020) 57:279–83. 10.1016/j.jcrc.2020.03.005 32173110 PMC7270792

[21] Touret F de Lamballerie X. Of chloroquine and COVID-19. Antiviral Res. (2020) 177:104762. 10.1016/j.antiviral.2020.104762 32147496 PMC7132364

[22] Sun D. Remdesivir for treatment of COVID-19: combination of pulmonary and IV administration may offer aditional benefit. AAPS J. (2020) 22:77. 10.1208/s12248-020-00459-8 32458279 PMC7250281

[23] de Wit E Feldmann F Cronin J Jordan R Okumura A Thomas T. Prophylactic and therapeutic remdesivir (GS-5734) treatment in the rhesus macaque model of MERS-CoV infection. Proc Natl Acad Sci USA. (2020) 117:6771–6. 10.1073/pnas.1922083117 32054787 PMC7104368

[24] Wang Y Zhang D Du G Du R Zhao J Jin Y. Remdesivir in adults with severe COVID-19: a randomised, double-blind, placebo-controlled, multicentre trial. Lancet. (2020) 395:1569–78. 10.1016/S0140-6736(20)31022-9 32423584 PMC7190303

[25] Kang CK Seong MW Choi SJ Kim TS Choe PG Song SH. *In vitro* activity of lopinavir/ritonavir and hydroxychloroquine against severe acute respiratory syndrome coronavirus 2 at concentrations achievable by usual doses. Korean J Intern Med. (2020) 35:782–7. 10.3904/kjim.2020.157 32460458 PMC7373950

[26] Rawizza HE Darin KM Oladokun R Brown B Ogunbosi B David N. Safety and efficacy of rifabutin among HIV/TB-coinfected children on lopinavir/ritonavir-based ART. J Antimicrob Chemother. (2019) 74:2707–15. 10.1093/jac/dkz219 31139825 PMC6736350

[27] Mozzini C Girelli D The role of neutrophil extracellular traps in Covid-19: only an hypothesis or a potential new field of research? Thromb Res. (2020) 191:26–7. 10.1016/j.thromres.2020.04.031 32360977 PMC7184981

[28] Dunbar JP Sulpice R Dugon MM. The kiss of (cell) death: can venom-induced immune response contribute to dermal necrosis following arthropod envenomations? Clin Toxicol. (2019) 57:677–85. 10.1080/15563650.2019.1578367 30806093

[29] Péterfi O Boda F Szabó Z Ferencz E Bába L. Hypotensive snake venom components-a mini-Review. Molecules. (2019) 24:1–16. 10.3390/molecules24152778 31370142 PMC6695636

[30] Hermann K Ring J. The renin angiotensin system and hymenoptera venom anaphylaxis. Clin Exp Allergy. (1993) 23:762–9. 10.1111/j.1365-2222.1993.tb00364.x 10779307

[31] Sun Y Han M Shen Z Huang H Miao X. Anti-hypertensive and cardioprotective effects of a novel apitherapy formulation via upregulation of peroxisome proliferator-activated receptor-α and -γ in spontaneous hypertensive rats. Saudi J Biol Sci. (2018) 25:213–9. 10.1016/j.sjbs.2017.10.010 29472767 PMC5816011

[32] Kim CMH Apitherapy - bee venom therapy. In: Biotherapy - History, Principles and Practice. Heidelberg: Springer (2013). p. 77–112. 10.1007/978-94-007-6585-6_4

[33] Hauser RA Daguio M Wester D Hauser M Kirchman A Skinkis C Bee-venom therapy for treating multiple sclerosis: a clinical trial. Altern Complement Ther. (2001) 7:37–45. 10.1089/107628001300000714

[34] Alvarez-Fischer D Noelker C Vulinović F Grünewald A Chevarin C Klein C. Bee venom and its component apamin as neuroprotective agents in a parkinson disease mouse model. PLoS ONE. (2013) 84:e61700. 10.1371/journal.pone.0061700 23637888 PMC3630120

[35] Beck BF Bee venom therapy. Bee Venom Therapy. Graphic Publishing Company. (1981). p. 238 Available online at: https://www.cabdirect.org/cabdirect/abstract/19820213710

[36] Pfaar O Klimek L Jutel M Akdis C Bousquet J Akdis M. Handling of allergen immunotherapy in the COVID-19 pandemic: an ARIA-EAACI statement. Allergy. (2020) 75:1546–54. 10.1111/all.14336 32329930 PMC7264744

[37] Block J High risk COVID-19: potential intervention at multiple points in the COVID-19 disease process via prophylactic treatment with azithromycin or bee derived products. Preprints. (2020) 2020040013. 10.20944/preprints202004.0013.v1

[38] Muller U Thurnheer U Patrizzii R Spies J Hoigne R. Immunotherapy in bee sting hypersensitivity: bee venom versus whole body extract. Allergy. (1979) 34:369–78. 10.1111/j.1398-9995.1979.tb02006.x 546252

[39] Marichal T Starkl P Reber LL Kalesnikoff J Oettgen HC Tsai M. A beneficial role for immunoglobulin E in host defense against honeybee venom. Immunity. (2013) 39:963–75. 10.1016/j.immuni.2013.10.005 24210352 PMC4164235

[40] Johansen P Senti G Martinez Gomez JM Storni T Beust BR Wuthrich B. Toll-like receptor ligands as adjuvants in allergen-specific immunotherapy. Clin Exp Allergy. (2005) 35:1591–8. 10.1111/j.1365-2222.2005.02384.x 16393325

[41] Caramalho I Melo A Pedro E Barbosa MMP Victorino RMM Pereira Santos MC. Bee venom enhances the differentiation of human regulatory T cells. Allergy. (2015) 70:1340–5. 10.1111/all.12691 26179427

[42] Kim H Keum DJ Kwak JW Chung H-S Bae H. Bee venom phospholipase a2 protects against acetaminophen-induced acute liver injury by modulating regulatory T cells and IL-10 in mice. PLoS ONE. (2014) 9:e114726. 10.1371/journal.pone.0114726 25478691 PMC4257707

[43] Pereira-Santos MC Baptista AP Melo A Alves RR Soares RS Pedro E. Expansion of circulating Foxp3+CD25bright CD4 + T cells during specific venom immunotherapy. Clin Exp Allergy. (2008) 38:291–7. 10.1111/j.1365-2222.2007.02887.x 18070166

[44] Lee G Bae H. bee venom phospholipase A2: yesterday's enemy becomes today's friend. Toxins. (2016) 8:48. 10.3390/toxins8020048 26907347 PMC4773801

[45] Yang J Zheng Y Gou X Pu K Chen Z Guo Q. Prevalence of comorbidities and its effects in patients infected with SARS-CoV-2: a systematic review and meta-analysis. Int J Infect Dis. (2020) 94:91–5. 10.1016/j.ijid.2020.03.017 32173574 PMC7194638

[46] Taketomi Y Ueno N Kojima T Sato H Murase R Yamamoto K. Mast cell maturation is driven via a group III phospholipase A 2-prostaglandin D2-DP1 receptor paracrine axis. Nat Immunol. (2013) 14:554–63. 10.1038/ni.2586 23624557 PMC4065307

[47] Lambeau G Lazdunski M. Receptors for a growing family of secreted phospholipases A2. Trends Pharmacol Sci. (1999) 20:162–70. 10.1016/S0165-6147(99)01300-0 10322502

[48] Park S Baek H Jung KH Lee G Lee H Kang GH. Bee venom phospholipase A2 suppresses allergic airway inflammation in an ovalbumin-induced asthma model through the induction of regulatory T cells. Immun Inflamm Dis. (2015) 3:386–97. 10.1002/iid3.76 26734460 PMC4693726

[49] Zahirović A Luzar J Molek P Kruljec N Lunder M. Bee venom immunotherapy: current status and future directions. Clin Rev Allergy Immunol. (2020) 58:326–41. 10.1007/s12016-019-08752-x 31240545

[50] Bellinghausen I Metz G EnkA H Christmann S Knop J Saloga J. Insect venom immunotherapy induces interleukin-10 production and a Th2-to-Th1 shift, and changes surface marker expression in venom-allergic subjects. Eur J Immunol. (1997). 27:1131–9. 10.1002/eji.1830270513 9174602

[51] ErŽen R Košnik M Šilar M Korošec P. Basophil response and the induction of a tolerance in venom immunotherapy: along term sting challenge study. Allergy. (2012) 67:822–30. 10.1111/j.1398-9995.2012.02817.x 22469017

[52] Jutel M Pichler WJ Skrbic D Urwyler A Dahinden C Müller U. Bee venom immunotherapy results in decrease of IL-4 and IL-5 and increase of IFN-gamma secretion in specific allergen-stimulated Tcell cultures. J Immunol. (1995) 154:4187–94. Available online at: https://www.jimmunol.org/content/154/8/4187.long 7706753

[53] Jakob T Rauber MM Perez-Riverol A Spillner E Blank S. The honeybee venom major allergen Api m 10 (Icarapin) and its role in diagnostics and treatment of hymenoptera venom allergy. Curr Allergy Asthma Rep. (2020) 20:48. 10.1007/s11882-020-00943-3 32548726 PMC7297703

[54] Shen L Lee JH Joo JC Park SJ Song J. Bee venom acupuncture for shoulder pain: a systematic review and meta-analysis of randomized controlled trials. J Pharmacopuncture. (2020) 23:44–53. 10.3831/KPI.2020.23.008 32685232 PMC7338706

[55] Bramwell VW Somavarapu S Outschoorn I AH. Adjuvant action of melittin following intranasal immunisation with tetanus and diphtheria toxoids. J Drug Target. (2003) 11:525–30. 10.1080/10611860410001670080 15203921

[56] Memariani H Memariani M Moravvej H Shahidi-Dadras M. Melittin: a venom-derived peptide with promising anti-viral properties. Eur J Clin Microbiol Infect Dis. (2020) 39:5–17. 10.1007/s10096-019-03674-0 31422545 PMC7224078

[57] Pucca MB Cerni FA Oliveira IS Jenkins TP Argemí L Sørensen C V. Bee updated: current knowledge on bee venom and bee envenoming therapy. Front Immunol. (2019) 10:2090. 10.3389/fimmu.2019.02090 31552038 PMC6743376

[58] Roy A Bharadvaja N Venom-derived bioactive compounds as potential anticancer agents: a review. Int J Pept Res Ther. (2020) 10.1007/s10989-020-10073-z

[59] An WW Gong XF Wang MW Tashiro S Onodera S Ikejima T. Norcantharidin induces apoptosis in HeLa cells through caspase, MAPK and mitochondrial pathways. Acta Pharmacol Sin. (2004) 25:1502–08. Available online at: http://www.chinaphar.com/article/view/8413/9071 15525474

[60] Cho H-J Jeong Y-J Park K-K Park Y-Y Chung I-K Lee K-G. Bee venom suppresses PMA-mediated MMP-9 gene activation via JNK/p38 and NF-κB-dependent mechanisms. J Ethnopharmacol. (2010) 127:662–8. 10.1016/j.jep.2009.12.007 19969058

[61] Baek YH Huh JE Lee JD Choi DY Park DS. Antinociceptive effect and the mechanism of bee venom acupuncture (Apipuncture) on inflammatory pain in the rat model of collagen-induced arthritis: mediation by α2-Adrenoceptors. Brain Res. (2006) 1073–1074:305–10. 10.1016/j.brainres.2005.12.086 16457792

[62] Choi J Jeon C Lee J Jang J Quan F Lee K. Suppressive effects of bee venom acupuncture on paclitaxel-induced neuropathic pain in rats: mediation by spinal α2-adrenergic receptor. Toxins. (2017) 9:351. 10.3390/toxins9110351 29088102 PMC5705966

[63] Chirumbolo S Zanoni G Ortolani R Vella A. *In vitro* biphasic effect of honey bee venom on basophils from screened healthy blood donors. Allergy Asthma Immunol Res. (2011) 3:58. 10.4168/aair.2011.3.1.58 21217927 PMC3005321

[64] Gu H Kim W-H An H Kim J Gwon M Han SM. Therapeutic effects of bee venom on experimental atopic dermatitis. Mol Med Rep. (2018) 18:3711–8. 10.3892/mmr.2018.9398 30132547 PMC6131226

[65] Bostanci N Belibasakis GN. Porphyromonas gingivalis: an invasive and evasive opportunistic oral pathogen. FEMS Microbiol Lett. (2012) 333:1–9. 10.1111/j.1574-6968.2012.02579.x 22530835

[66] Putz T Ramoner R Gander H Rahm A Bartsch G Thurnher M. Antitumor action and immune activation through cooperation of bee venom secretory phospholipase A2 and phosphatidylinositol-(3,4)-bisphosphate. Cancer Immunol Immunother. (2006) 55:1374–83. 10.1007/s00262-006-0143-9 16485125 PMC11030777

[67] Son D Lee J Lee Y Song H Lee C Hong J. Therapeutic application of anti-arthritis, pain-releasing, and anti-cancer effects of bee venom and its constituent compounds. Pharmacol Ther. (2007) 115:246–70. 10.1016/j.pharmthera.2007.04.004 17555825

[68] Palm NW Rosenstein RK Medzhitov R. Allergic host defences. Nature. (2012) 484:465–72. 10.1038/nature11047 22538607 PMC3596087

[69] Jilek S Barbey C Spertini F Corthésy B. Antigen-independent suppression of the allergic immune response to bee venom phospholipase A 2 by DNA vaccination in CBA/J mice. J Immunol. (2001) 166:3612–21. 10.4049/jimmunol.166.5.3612 11207323

[70] Palm NW Rosenstein RK Yu S Schenten DD Florsheim E Medzhitov R. Bee venom phospholipase A2 induces a primary type 2 response that is dependent on the receptor ST2 and confers protective immunity. Immunity. (2013) 39:976–85. 10.1016/j.immuni.2013.10.006 24210353 PMC3852615

[71] An HJ Lee WR Kim KH Kim JY Lee SJ Han SM. Inhibitory effects of bee venom on propionibacterium acnes-induced inflammatory skin disease in an animal model. Int J Mol Med. (2014) 34:1341–8. 10.3892/ijmm.2014.1933 25215662

[72] Rizk JG Kalantar-Zadeh K Mehra MR Lavie CJ Rizk Y Forthal DN Pharmaco-Immunomodulatory therapy in COVID-19. Drugs. (2020) 80:1267–92. 10.1007/s40265-020-01367-z 32696108 PMC7372203

[73] Babon A Almunia C Boccaccio C Beaumelle B Gelb MH Ménez A. Cross-presentation of a CMV pp65 epitope by human dendritic cells using bee venom PLA 2 as a membrane-binding vector. FEBS Lett. (2005) 579:1658–64. 10.1016/j.febslet.2005.02.019 15757657

[74] Almunia C Bretaudeau M Held G Babon A Marchetti C Castelli FA. Bee venom phospholipase A2, a good “Chauffeur” for delivering tumor antigen to the MHC I and MHC II peptide-loading compartments of the dendritic cells: the case of NY-ESO-1. PLoS ONE. (2013) 8:1–17. 10.1371/journal.pone.0067645 23825678 PMC3688974

[75] Eltahir Saeed WS Gasim Khalil EA Immune response modifying effects of bee venom protein [Melittin]/Autoclaved L. donovani complex in CD1 Mice: the search for new vaccine adjuvants. J Vaccines Vaccin. (2017) 08:6–11. 10.4172/2157-7560.1000372

[76] Cherniack EP Govorushko S To bee or not to bee: the potential efficacy and safety of bee venom acupuncture in humans. Toxicon. (2018) 154:74–8. 10.1016/j.toxicon.2018.09.013 30268393

[77] Seo B-K Han K Kwon O Jo D-J Lee J-H. Efficacy of bee venom acupuncture for chronic low back pain: a randomized, double-blinded, sham-controlled trial. Toxins. (2017) 9:361. 10.3390/toxins9110361 29112155 PMC5705976

[78] Gu H Han SM Park K-K. therapeutic effects of apamin as a bee venom component for non-neoplastic disease. Toxins. (2020) 12:195. 10.3390/toxins12030195 32204567 PMC7150898

[79] Rady I Siddiqui IA Rady M Mukhtar H. Melittin, a major peptide component of bee venom, and its conjugates in cancer therapy. Cancer Lett. (2017) 402:16–31. 10.1016/j.canlet.2017.05.010 28536009 PMC5682937

[80] Son DJ Kang J Kim TJ Song HS Sung KJ Yun DY. Melittin, a major bioactive component of bee venom toxin, inhibits PDGF receptor beta-tyrosine phosphorylation and downstream intracellular signal transduction in rat aortic vascular smooth muscle cells. J Toxicol Environm Health Part A. (2007) 70:1350–5. 10.1080/15287390701428689 17654254

[81] Zhang S Liu Y Ye Y Wang XR Lin LT Xiao LY. Bee venom therapy: potential mechanisms and therapeutic applications. Toxicon. (2018) 148:64–73. 10.1016/j.toxicon.2018.04.012 29654868

[82] King TP Jim SY Wittkowski K. Inflammatory role of two venom components of yellow jackets (Vespula vulgaris): A mast cell degranulating peptide mastoparan and phospholipase A1. Int Arch Allergy Immunol. (2003) 131:25–32. 10.1159/000070431 12759486

[83] LaFerla FM Green KN Oddo S. Intracellular amyloid-beta in alzheimer's disease. Nat Rev Neurosci. (2007) 8:499–509. 10.1038/nrn2168 17551515

[84] Shkenderov S Koburova K. Adolapin-A newly isolated analgetic and anti-inflammatory polypeptide from bee venom. Toxicon. (1982) 20:317–21. 10.1016/0041-0101(82)90234-3 7080045

[85] Moreno M Giralt E. Three valuable peptides from bee and wasp venoms for therapeutic and biotechnological use: melittin, apamin and mastoparan. Toxins. (2015) 7:1126–50. 10.3390/toxins7041126 25835385 PMC4417959

[86] Bilò MB Bonifazi F. The natural history and epidemiology of insect venom allergy: clinical implications. Clin Exp Allergy. (2009) 39:1467–76. 10.1111/j.1365-2222.2009.03324.x 19622088

[87] Antonicelli L Bilò MB Bonifazi F. Epidemiology of hymenoptera allergy. Curr Opin Allergy Clin Immunol. (2002) 2:341–6. 10.1097/00130832-200208000-00008 12130949

[88] Mingomataj EÇ Bakiri AH. Episodic hemorrhage during honeybee venom anaphylaxis: potential mechanisms. J Investig Allergol Clin Immunol. (2012) 22:237–44. Available online at: http://www.jiaci.org/issues/vol22issue4/vol22issue04-1.html 22812191

[89] White J Venomous Animals: Clinical Toxinology. In: EXS (2010) p. 233–91. Available online at: http://www.ncbi.nlm.nih.gov/pubmed/20358686 10.1007/978-3-7643-8338-1_7 20358686

[90] Vetter RS Visscher PK. Bites and stings of medically important venomous arthropods. Int J Dermatol. (1998) 37:481–96. 10.1046/j.1365-4362.1998.00455.x 9679688

[91] Lima PR de Brochetto-Braga MR Hymenoptera venom review focusing on *Apis mellifera*. J Venom Anim Toxins Incl Trop Dis. (2003) 9:149–62. 10.1590/S1678-91992003000200002

[92] Golden DBK. Epidemiology of allergy to insect venoms and stings. Allergy Asthma Proc. (1989) 10:103–7. 10.2500/108854189778960964 2661327

[93] Stoevesandt J Sturm GJ Bonadonna P Oude Elberink JNG Trautmann A. Risk factors and indicators of severe systemic insect sting reactions. Allergy Eur J Allergy Clin Immunol. (2020) 75:535–45. 10.1111/all.13945 31194889

[94] Reber LL Hernandez JD Galli SJ. The pathophysiology of anaphylaxis. J Allergy Clin Immunol. (2017) 140:335–48. 10.1016/j.jaci.2017.06.003 28780941 PMC5657389

[95] Chen J Guan S-M Sun W Fu H. Melittin, the major pain-producing substance of bee venom. Neurosci Bull. (2016) 32:265–72. 10.1007/s12264-016-0024-y 26983715 PMC5563768

[96] Abd El-Wahed AA Khalifa SAM Sheikh BY Farag MA Saeed A Larik FA Bee Venom Composition: From Chemistry to Biological Activity. Stud Nat Prod Chem. (2019) 60:459–84. 10.1016/B978-0-444-64181-6.00013-9

[97] Stone KD Prussin C Metcalfe DD. IgE, mast cells, basophils, and eosinophils. J Allergy Clin Immunol. (2010) 125:S73–80. 10.1016/j.jaci.2009.11.017 20176269 PMC2847274

[98] Schumacher M Tveten M Egen N. Rate and quantity of delivery of venom from honeybee stings. J Allergy Clin Immunol. (1994). 93:831–5. 10.1016/0091-6749(94)90373-5 8182223

[99] Toledo LFM Moore DCBC Caixeta DMDL Salú MDS Farias CVB Azevedo ZMA. Multiple bee stings, multiple organs involved: a case report. Rev Soc Bras Med Trop. (2018). 51:560–2. 10.1590/0037-8682-0341-2017 30133647

[100] Rajendiran C Puvanalingam A Thangam D Ragunanthanan S Ramesh D Venkatesan S. Stroke after multiple bee sting. J Assoc Physicians India. (2012) 60:122–4. 22715562

[101] Bhalotia S Kumar NR Kaur J Devi A Honey bee venom and its composition: focusing on different apis species-a review. J Basic Appl Eng Res. (2016) 3:96–8. Available online at: https://www.krishisanskriti.org/vol_image/10Jun2016100659z47%20%20%20%20%20%20%20%20%20%20%20Anita%20Devi%202%20%20%20%20%20%20%20%20%20%20%2096-98.pdf

[102] Hossen MS Gan SH Khalil MI Melittin, a potential natural toxin of crude bee venom: probable future arsenal in the treatment of diabetes mellitus. J Chem. (2017) 2017:1–7. 10.1155/2017/4035626

[103] Wehbe R Frangieh J Rima M El Obeid D Sabatier J-M Fajloun Z. Bee venom: overview of main compounds and bioactivities for therapeutic interests. Molecules. (2019) 24:2997. 10.3390/molecules24162997 31430861 PMC6720840

[104] Mokosuli YS Repi RA Worang RL Mokosuli C Semuel Y Potential antioxidant and anticancer effect of apis dorsata binghami crude venom from minahasa, north sulawesi. J Entomol Zool Stud JEZS. (2017) 112:112–9. Available online at: https://www.entomoljournal.com/archives/?year=2017&vol=5&issue=2&ArticleId=1581

[105] Bachmayer H Kreil G Suchanek G Synthesis of promelittin and melittin in the venom gland of queen and worker bees: patterns observed during maturation. J Insect Physiol. (1972) 18:1515–21. 10.1016/0022-1910(72)90230-2

[106] Abusabbah M Hong Lau W Mahmoud ME Salih AM Omar D Prospects of using carbohydrates as supplemented-diets and protein rich mixture as alternative-diet to improve the quality of venom produced by Apis cerana L. J Entomol Zool Stud. (2016) 4:23–6.

[107] Abdela N Jilo K Bee venom and its therapeutic values: a review. Adv Life Sci Technol. (2016) 44:18–22. Available online at: https://www.iiste.org/Journals/index.php/ALST/article/view/30404/31249

[108] Owen MD Pfaff LA. Melittin synthesis in the venom system of the honey bee (Apis mellifera L). Toxicon. (1995) 33:1181–8. 10.1016/0041-0101(95)00054-P 8585088

[109] Leandro LF Mendes CA Casemiro LA Vinholis AHC Cunha WR Almeida R de. Antimicrobial activity of apitoxin, melittin and phospholipase A2 of honey bee (Apis mellifera) venom against oral pathogens. An Acad Bras Cienc. (2015) 87:147–55. 10.1590/0001-3765201520130511 25806982

[110] Uddin MB Lee B-H Nikapitiya C Kim J-H Kim T-H Lee H-C. Inhibitory effects of bee venom and its components against viruses in vitro and in vivo. J Microbiol. (2016) 54:853–66. 10.1007/s12275-016-6376-1 27888461 PMC7091203

[111] Jung GB Huh J-E Lee H-J Kim D Lee G-J Park H-K. Anti-cancer effect of bee venom on human MDA-MB-231 breast cancer cells using Raman spectroscopy. Biomed Opt Express. (2018) 9:5703 10.1364/BOE.9.005703 30460157 PMC6238932

[112] Sabaratnam V Gurunathan S Raman J Abd Malek SN John P Green synthesis of silver nanoparticles using Ganoderma neo-japonicum Imazeki: a potential cytotoxic agent against breast cancer cells. Int J Nanomedicine. (2013) 8:4399 10.2147/IJN.S51881 24265551 PMC3833323

[113] Hong J Lu X Deng Z Xiao S Yuan B Yang K. How melittin inserts into cell membrane: conformational changes, inter-peptide cooperation, and disturbance on the membrane. Molecules. (2019) 24:1775. 10.3390/molecules24091775 31067828 PMC6539814

[114] Liu S Yu M He Y Xiao L Wang F Song C. Melittin prevents liver cancer cell metastasis through inhibition of the Rac1-dependent pathway. Hepatology. (2008) 47:1964–73. 10.1002/hep.22240 18506888

[115] Gupta T Gupta SK. Potential adjuvants for the development of a SARS-CoV-2 vaccine based on experimental results from similar coronaviruses. Int Immunopharmacol. (2020) 86:106717. 10.1016/j.intimp.2020.106717 32585611 PMC7301105

[116] Okba NM Raj VS Haagmans BL. Middle East respiratory syndrome coronavirus vaccines: current status and novel approaches. Curr Opin Virol. (2017) 23:49–58. 10.1016/j.coviro.2017.03.007 28412285 PMC7102752

[117] Vijay R Hua X Meyerholz DK Miki Y Yamamoto K Gelb M. Critical role of phospholipase A2 group IID in age-related susceptibility to severe acute respiratory syndrome-CoV infection. J Exp Med. (2015) 212:1851–68. 10.1084/jem.20150632 26392224 PMC4612096

[118] Mousavizadeh L Ghasemi S. Genotype and phenotype of COVID-19: Their roles in pathogenesis. J Microbiol Immunol Infect. (2020) 2020:5. 10.1016/j.jmii.2020.03.022 32265180 PMC7138183

[119] Dawood AA. Mutated COVID-19 may foretell a great risk for mankind in the future. New Microbes New Infect. (2020) 35:100673. 10.1016/j.nmni.2020.100673 32292587 PMC7129032

[120] de Haan CAM Kuo L Masters PS Vennema H Rottier PJM. Coronavirus particle assembly: primary structure requirements of the membrane protein. J Virol. (1998) 72:6838–50. 10.1128/JVI.72.8.6838-6850.1998 9658133 PMC109893

[121] Fenard D Lambeau G Valentin E Lefebvre J-C Lazdunski M Doglio A. Secreted phospholipases A2, a new class of HIV inhibitors that block virus entry into host cells. J Clin Invest. (1999) 104:611–8. 10.1172/JCI6915 10487775 PMC408539

[122] Soman NR Baldwin SL Hu G Marsh JN Lanza GM Heuser JE. Molecularly targeted nanocarriers deliver the cytolytic peptide melittin specifically to tumor cells in mice, reducing tumor growth. J Clin Invest. (2009) 119:2830–42. 10.1172/JCI38842 19726870 PMC2735896

[123] Youngren-Ortiz SR Chougule MB Morris KR Development and evaluation of siRNA loaded gelatin nanocarriers for the treatment of asthma. Dissertations and Theses. University of Hawaii at Hilo. (2016) Available online at: https://dspace.lib.hawaii.edu/handle/10790/2758

[124] Yang W Hu F Xu X. Bee venom and SARS-CoV-2. Toxicon. (2020) 181:69–70. 10.1016/j.toxicon.2020.04.105 32360140 PMC7190514

[125] Balozet L Bücherl W Klobusitzky D De Valle JR Halstead BW Mcmichael DF Contributors to this volume venomous animals. (1971) 3:1–459.

[126] Casewell NR Wüster W Vonk FJ Harrison RA Fry BG. Complex cocktails: the evolutionary novelty of venoms. Trends Ecol Evol. (2013) 28:219–29. 10.1016/j.tree.2012.10.020 23219381

[127] Garraud O Hozzein WN Badr G. Wound healing: time to look for intelligent, ‘natural’ immunological approaches? BMC Immunol. (2017) 18:23. 10.1186/s12865-017-0207-y 28681702 PMC5499069

[128] Ali M Studies on bee venom and its medical uses. Int J Adv Res. (2012) 1:1–15. Available online at: http://www.ijoart.org/docs/Studies-on-Bee-Venom-and-Its-Medical-Uses.pdf

[129] Seif F Khoshmirsafa M Aazami H Mohsenzadegan M Sedighi G Bahar M. The role of JAK-STAT signaling pathway and its regulators in the fate of T helper cells. Cell Commun Signal. (2017) 15:23. 10.1186/s12964-017-0177-y 28637459 PMC5480189

[130] Dams D Briers Y. Enzybiotics: enzyme-based antibacterials as therapeutics. Adv Exp Med Biol. (2019) 1148:233–53. 10.1007/978-981-13-7709-9_11 31482502

[131] Boens S Szekér K Eynde Van A Bollen M Phosphatase Modulators. In: Millán JL editor. Methods in Molecular Biology. Totowa, NJ: Humana Press (2013). p.271–81. 10.1007/978-1-62703-562-0_16 23860659

[132] Szulc P Bauer DC Biochemical markers of bone turnover in osteoporosis. In: Osteoporosis. Elsevier (2013). p. 1573–610. 10.1016/B978-0-12-415853-5.00067-4 Available online at: https://www.sciencedirect.com/science/article/pii/B9780124158535000674?via%3Dihub

[133] Murakami M Nakatani Y Atsumi G Inoue K Kudo I. Regulatory functions of phospholipase A2. Crit Rev Immunol. (2017) 37:121–79. 10.1615/CritRevImmunol.v37.i2-6.20 29773019

[134] Stahelin R V Chapter 8 - Phospholipid Catabolism. In: Ridgway ND McLeod L editors. Lipoproteins and Membranes (Sixth Edition) RSBT-B. Boston: Elsevier (2016) p. 237–57. 10.1016/B978-0-444-63438-2.00008-0

[135] Connolly TM Lawing WJ Majerus PW. Protein kinase C phosphorylates human platelet inositol trisphosphate 5′-phosphomonoesterase, increasing the phosphatase activity. Cell. (1986) 46:951–8. 10.1016/0092-8674(86)90077-2 3019558

[136] Brás NF Santos-Martins D Fernandes PA Ramos MJ Mechanistic pathway on human α-glucosidase maltase-glucoamylase Unveiled by QM/MM calculations. J Phys Chem B. (2018) 122:3889–99. 10.1021/acs.jpcb.8b01321 29548257

[137] Holtsberg FW Ozgur LE Garsetti DE Myers J Egan RW Clark MA. Presence in human eosinophils of a lysophospholipase similar to that found in the pancreas. Biochem J. (1995) 309:141–4. 10.1042/bj3090141 7619049 PMC1135811

[138] Karamitros CS Konrad M. Human 60-kDa lysophospholipase contains an N-terminal l-Asparaginase domain that is allosterically regulated by l-Asparagine. J Biol Chem. (2014) 289:12962–75. 10.1074/jbc.M113.545038 24657844 PMC4036312

[139] Soliman C Eastwood S Truong VK Ramsland PA Elbourne A. The membrane effects of melittin on gastric and colorectal cancer. PLoS ONE. (2019) 14:e0224028. 10.1371/journal.pone.0224028 31622415 PMC6797111

[140] Issa MF Tuboly G Kozmann G Juhasz Z Automatic ECG artefact removal from EEG signals. Meas Sci Rev. (2019) 19:101–8. 10.2478/msr-2019-0016

[141] Elieh Ali Komi D Shafaghat F Zwiener RD. Immunology of bee venom. Clin Rev Allergy Immunol. (2018) 54:386–96. 10.1007/s12016-017-8597-4 28105558

